# A Comparison of the Effect of Facemasks on Perceived Breathability and Air Quality during Daily Activities and Indoor Exercises

**DOI:** 10.3390/ijerph20054144

**Published:** 2023-02-25

**Authors:** Lai-Yin Qin

**Affiliations:** Academy of Visual Arts, School of Creative Arts, Hong Kong Baptist University, Hong Kong, China; annaqin@hkbu.edu.hk

**Keywords:** face mask, perceived comfort, perceived breathability, perceived air quality, indoor exercises, airborne transmission, COVID-19 pandemic, self-controlled case series

## Abstract

Transmission of COVID-19 occurs predominantly through respired droplets and aerosols containing the SARS-CoV-2 virus. As a solution, face masks have been used to protect against infection. Wearing face masks during indoor exercises is essential to prevent the spread of virus-containing respiratory droplets and aerosols. However, previous studies have not investigated all elements, including the users’ perceived breathability (PB) and perceived air quality (PAQ) when wearing a face mask during indoor exercises. The current study aimed to assess users’ perceived comfort (PC) of face masks based on assessment criteria of PB and PAQ during moderate to vigorous exercises, and compare them with those during normal daily activities. Data on PC, PB, and PAQ were collected from an online questionnaire survey from 104 participants doing regular moderate to vigorous exercises. Within-subjects comparison with self-controlled case series design was performed to compare PC, PB, and PAQ between wearing face masks during exercises and daily activities. Results showed that the degree of dissatisfaction with PC, PB, and PAQ while wearing face masks and performing indoor exercises is higher than when performing daily activities (*p* < 0.05). The significance of the study implies that masks comfortable for daily activities may not remain the same during moderate to vigorous exercises, especially during indoor exercises.

## 1. Introduction

The coronavirus disease-19 (COVID-19) pandemic, caused by coronavirus 2 (SARS-CoV-2), has led to the most significant trans-century global public health emergency, resulting in 643.6 million infection cases and 6.6 million deaths worldwide [1]. Transmission of COVID-19 occurs predominantly through respired droplets and aerosols containing the SARS-CoV-2 virus [2,3,4]. Droplets and aerosols with pathogens are spread to the air through breathing, talking, coughing, or sneezing by infected individuals and inhaled by close-contacts into their respiratory system. Therefore, face masks have been recommended by the World Health Organization (WHO) as a critical measure for reducing infection rates. Face masks work by blocking droplets and aerosols from infected patients and filtering particles out of the air to protect healthy people against airborne viruses, which are not limited to SARS-CoV-2 [5,6]. Epidemiologic studies have shown evidence that using face masks effectively prevents contracting and transmitting COVID-19 [7,8,9].

Before the COVID-19 pandemic, the fitness industry was steadily growing globally with an increasing number of members of indoor leisure sports and fitness centers [10,11]. It is widely known that regular physical activity can improve physical and mental health as well as quality of life. However, previous studies have demonstrated that vigorous exercise generated more droplets and aerosols than at rest [12,13]. The increased ventilation rates are reflected during conversational speech as well as increased efforts during exercise [12]. In Hong Kong, an indoor gym was traced as a possible source of an outbreak in March 2021 [13]. Wearing face masks when exercising indoor is essential to prevent the spread of infectious respiratory droplets and aerosols. To ensure solution effectiveness, the indoor and outdoor mask mandate in Hong Kong commenced on 23 July 2020 [14,15].

The intensity of exercises can be classified into moderate and vigorous levels. Both moderate and vigorous activities will raise the heart rate, resulting in faster and warmer breaths. Working at a moderate intensity level, the participant can still talk but not sing [16]. Vigorous intensity activity makes one breathe harder and faster; working at this level, the participants cannot say more than a few words without pausing for breath [16]. Research evidence exists that “wearing a face mask during vigorous exercise had no discernable detrimental effect on blood or muscle oxygenation” [16] and exercise performance in young, healthy participants [17,18,19,20,21,22,23,24]. Though the biophysical parameters were not affected, many participants still complained of discomfort wearing face masks while exercising. Common complaints regarding discomfort were decreased ease of breath and unpleasant odors from the face mask during moderate to vigorous exercises indoors. There are very few studies [17,18,19,20,21,22,23,24] investigating the users’ perceived discomfort with significant findings. Three studies [21,22,23,24] reported that wearing a face mask when engaging in exercises produced a feeling of discomfort, while four other studies [17,18,19,20] concluded no significant difference in perceived discomfort during exercises and regular activities. These studies included a small sample size, only 6–23 healthy candidates [17,18,19,20,21,22,23,24]. 

Quantitative measurement of ease of breath through a material is referred as breathability. The breathability of a face mask is measured by “the pressure drop between the two sides of a mask as air flows through it at a rate similar to that during normal breathing” and is tested at a specific flow rate across a specified material surface area [25]. In this study, ease of breath refers to the “perceived breathability (PB)” of face masks, while the perceived air quality (PAQ) used for assessing environmental air freshness [26] has been adapted to assess the air freshness within masks. 

The purpose of the current study was to find empirical evidence regarding the perceived comfort (PC) of face masks based on criteria including PB and PAQ during moderate to vigorous exercises, and comparing these results with findings about usage during daily activities.

## 2. Materials and Methods

### 2.1. Study Design

This study utilized a self-controlled case series design [27,28] in which a participant acted as his/her own control for comparison issues, thereby minimizing potential confounders such as age, gender, education level, etc. [27,28].

An online questionnaire titled “Mask-Wearing Knowledge and Sensory Quality in Hong Kong during the COVID-19 Pandemic” was distributed through a surveying platform named QuestionPro [29]. This online survey investigates the overall mask-wearing experience in Hong Kong, highlighting participants’ PC, PB, and PAQ with the mask of their daily choice. Within-subjects comparison was used for comparing PC, PB, and PAQ when wearing face masks during daily activities and vigorous exercises. Human ethics approval was granted from the Hong Kong Baptist University (approval reference number REC21-22/0219) prior to commencing the study. 

### 2.2. Participants

All participants were informed and signed a “Consent to Participate in Research” form before answering the questionnaire. In total, 104 participants were recruited online at a community sports center in Hong Kong. Inclusion and exclusion criteria were specified and included in the short questionnaire for pre-screening, and 104 participants who satisfied both inclusion and exclusion criteria were recruited in this study for analysis. The inclusion criteria were (1) age between 18 to 64 years old, (2) both genders, (3) doing moderate to vigorous exercises two or more times per week, and (4) wearing face masks daily and during indoor exercises. The exclusion criteria included individuals with (1) poor olfactory function and (2) smoking habits.

The sample size was estimated based on preliminary study findings after performing the same survey on 20 subjects, and the greatest standard deviation (SD) of differences in all outcome measurements was 1.445. The sample size was calculated with the threshold probability for rejecting the null hypothesis type I error rate α (two-tailed) = 0.05, the probability of failing to reject the null hypothesis under the alternative hypothesis type II error rate β = 10%, and effect size = 0.5. This resulted in a required sample size of 91 [30]. 

### 2.3. Methods

An online survey consisting of four parts was conducted for each participant. Part one included the consent and agreement of research data use, with the participant screening questions to ensure matching of the inclusion and exclusion criteria. Part two recorded the demographic information with general information on the participant’s characteristics, such as gender, age, and education level. Parts three and four consisted of key questions that collected semi-quantitative data on PC, PB, and PAQ during daily activities and exercising, respectively. Data of perceived comfort included (1) PC in general, (2) PB rated with the extent of disturbance of breath, and (3) PAQ measured with the perceived unpleasant odor of the air through face mask. Questions within these two survey sections included the type of face masks being worn, satisfactory levels towards the choice, whether any unpleasant smells were noticed, and the level of disturbance to the activity. Participants reported PC based on the 5-point bipolar Likert scale [31,32]: −2: very unsatisfied; −1: unsatisfied; 0: neutral; +1: satisfied; +2: very satisfied. Disturbance of breath and perceived unpleasant odor were rated with a 5-point unipolar Likert scale [31,32] ranging from 5 as never; 4 as seldom; 3 as sometimes; 2 as often; and 1 as always. 

### 2.4. Statistical Analysis

Data collected from all 104 participants have been included in the analysis. The analysis was conducted in IBM SPSS 25 Statistic (IBM Corp, Armonk, NY, USA). Descriptive statistics were reported for all participants. 

Paired *t*-test was used for within-subject comparison of PC, PB, and PAQ during daily life and during the period of exercise. A value of *p* < 0.05 is considered statistically significant.

## 3. Results

### 3.1. Participant Characteristics

**Figure 1:**
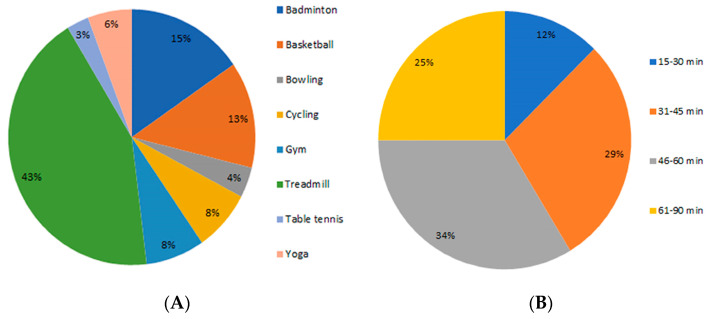
Distribution of 104 candidates according to (**A**) type of exercise and (**B**) duration of exercise.

All 104 eligible participants from Hong Kong completed the questionnaire for data analysis. The general information of the participants is summarized in Table 1. There were 61 males (58.7%) and 43 females (41.3%) aged between 18–64 years old, with the majority (76 participants 73.1%) between 25–44 years of age. Most participants (71, 68.3%) had a bachelor’s degree, 17 completed secondary school, and another 16 had a post-graduate education. All participants participated in regular moderate/vigorous exercises twice or more per week in indoor sports centers. The most popular exercise was running on the treadmill (45, 43.3%); other exercises included badminton, basketball, gym, cycling, yoga, bowling, and table tennis, in order of popularity. The duration of the exercises ranged from 15–90 min (Figure 1).

### 3.2. Types of Face Masks

Surgical face masks were used by most participants (76, 73.1%); other types of masks included N95, KF94, and reusable cotton masks (Table 2).

### 3.3. Descriptive Analysis of PC, PB, and PAQ

#### 3.3.1. PC of Wearing Face Masks

Table 3 summarizes the PC of wearing face masks in general. Twenty-nine participants (27.9%) rated PC of wearing face masks as unsatisfied or very unsatisfied during daily activities. The number of unsatisfied or very unsatisfied participants increased to 67 (64.4%) during indoor exercising.

#### 3.3.2. PB of Wearing Face Masks

Table 4 summarizes the PB by measuring the extent of perceived disturbance in respiration. Twenty-nine participants (27.9%) rated “often” or “always” perceiving disturbance while breathing during daily activities. This number increased to 68 (65.4%) during indoor exercising.

#### 3.3.3. PAQ of Wearing Face Masks

Table 5 summarizes the PAQ by measuring the perceived unpleasant breath odor and sweat odor. Twenty-three participants (22.1%) perceived unpleasant breath odor and another twenty-one participants (20.2%) perceived unpleasant sweat odor from the face masks “often” or “always” during daily activities. These numbers increased to 53 (51.0%) and 51 (49.0%) during indoor exercising, respectively.

### 3.4. Comparison of PC, PB, and PAQ during Daily Life and the Period of Exercise

**Figure 2:**
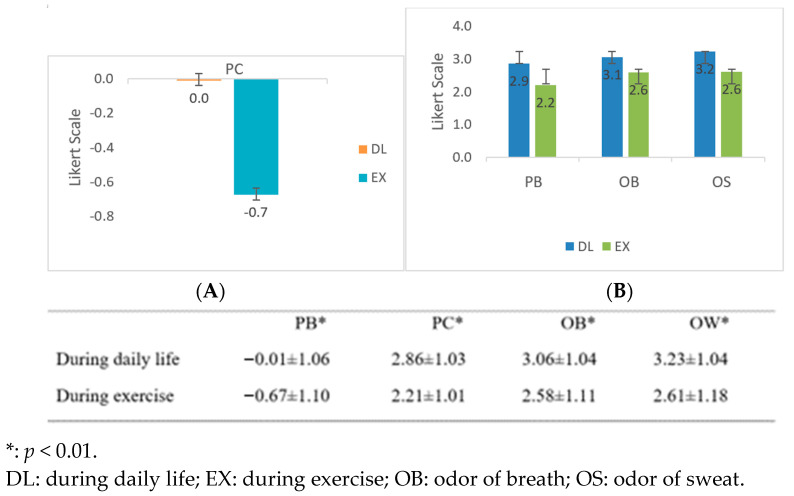
The means and SD of (**A**) PC, (**B**) PB, and PAQ (OB and OS).

The mean and standard deviation (SD) of PC, PB, and PAQ by measuring the perceived unpleasant odor of breath (OB) and odor of sweat (OS) during daily life and the period of exercise have been presented in Figure 2 and compared with a paired *t*-test. There is a significant difference between the perceived sensations during the period of exercise and daily activity in PC (*p* < 0.01), PB (*p* < 0.01), and PAQ to be presented by unpleasant OB (*p* < 0.01) and OS (*p* < 0.01).

## 4. Discussion

Wearing masks can protect the user by filtering the infected droplets based on the mask’s filtration effectiveness. The mask protects users physically; however, it can cause possible psychological anxieties due to the perceived discomforts suggested in the hypothesis of this study. This research concerned users’ perceived comfort, perceived breathability, and perceived air quality of face masks during moderate to vigorous exercises and compared them with findings regarding usage during daily activities. Key findings of this survey are that when participants perform moderate to vigorous indoor exercises, the number of dissatisfactions with PC, PB, and PAQ of face masks are significantly higher compared to daily activities. The extents were well explained by differences for all parameters with self-controlled comparison at a statistical significance (*p* < 0.01). 

### 4.1. Breathability

The majority of participants (73.1%) in this study used surgical masks for both daily activities and indoor exercises. Other participants used N95, KF94, and several used cotton or other reusable masks. This survey was conducted in Hong Kong, and the quality of commercially available surgical, N95, and KD 94 masks satisfied the standard of material filtration efficiency and breathability [25]. Perceived breathability is the ease of breathing through a mask, while the pressure differential measures material breathability between the two sides of a mask as air flows through it at a volume and velocity similar to that of breathing during daily activities [33]. Moderate to vigorous exercises increased both the volume and velocity of air flow [30]. According to Darcy’s law [34], “the difference between the pressure on the upstream side of a porous material and the pressure on the downstream side is proportional to the face velocity of air through the material”. Thus, moderate and vigorous exercises increased the pressure differential between both sides of a mask and decreased the breathability. Mapelli et al. [19] reported a significant increase in the values of shortness of breath when wearing a face mask during exercise, although there was no significant difference in cardiopulmonary measurements. Findings by Mappeli et al. [19] are in agreement with our findings. Future research designed to test breathability and tissue oxygenation concurrently is beneficial to investigate the relationship between the two factors.

### 4.2. Perceived Air Quality

The study also confirms that more participants perceived an unpleasant odor during moderate and vigorous exercises than during daily activities. The source of the unpleasant odor is indicated to be from breath and sweat. This survey is the first study to provide research evidence that showed statistical significance in exercises increasing the unpleasant odor of the mask though many mask users found these phenomena. Hong et al. [23] have evaluated the odor in face mask as one of the multiple discomfort feelings of wearing face mask during exercises, and their finding was that exercise resulted in a significantly higher discomfort score. They did not report the odor score separately. 

A hypothesis attributed the unpleasant odors to the adhesion of respiratory particles on the mask. The bacteria in those particles may then grow and cause a distasteful odor. Orton et al. [12] carried out an experiment to compare respiratory particle emission rates at rest and while speaking or exercising. They found that “vigorous exercise and very vigorous exercise generated approximately four and eight times greater numbers of aerosol particles (based on median values) than breathing at rest”. This finding may be the reason for the vigorous exercises that increase the unpleasant odor of masks through a larger volume of aerosols landing on the mask’s inner surface. To this point, studies still have not drawn a direct relationship between the type and bacteria quantity in the aerosols and how they relate to the odor. A survey on self-perceived and self-reported breath odor and wearing face masks was conducted that showed a close relationship between odor and dental problems, smoking, alcohol drinking, taking medicine, etc., yet without showing laboratory findings from the masks with odors [35]. A recent study also showed that physical factors, e.g., heat transfer on the surface of the nasal mucosa, could also affect the perceived freshness of air [36].

## 5. Conclusions

In summary, the results of this study suggest that masks that are comfortable for daily activities may not remain the same during moderate to vigorous exercises, especially during indoor exercises. Face masks with new materials and designs are therefore desirable to achieve proper filtration efficiency, comfortable breathability, and less unpleasant odor for the purpose of exercise.

### 5.1. Implications

This study examines the overall experience of wearing masks daily for multi activities, contributing to possible improvements in mask and accessory designs to ease any unpleasant experience, especially odor problems. Face masks, as an original tool to prevent respiratory droplets from causing infection in surgery, pandemics, and other situations, often require long hours of wear. Increasing research findings in neuroscience suggest that olfactory stimulus plays a role in central nervous system function beyond that of smell [37,38]. Perceived comfort while wearing masks is essential for the user to maintain favorable psychological conditions [39]. 

### 5.2. Limitations and Future Study

There are some limitations to be considered while interpreting this study. The current study focused on showing evidence that there were differences in perceived comfort, perceived breathability, and perceived air quality during daily activities and during moderate to vigorous exercises. Personal and social characteristics such as age, gender, and educational level might have an effect on the outcome measures; hence, the functional capacity of perceiving comfort and breathability might vary with these factors. To eliminate the effect of personal and social factors on the outcome measurements, the researcher employed “self-controlled case series design” for this project, where the participant serves as his/her own control. This is used to investigate the transient effects of intermittent exposure on the onset of acute outcomes. The advantage of the self-controlled case series design is to minimize the influence of individual attribution factors on the outcome measures [27,28]. However, this retrospective questionnaire study has the disadvantage that it cannot eliminate the variation of independent variables, including the intensity and duration of daily activities and exercises. The intensity of exercises in this study ranged from bowling and table tennis which are of moderate intensity, to badminton and basketball which are of vigorous intensity [16]. The intensity of other exercises including cycling, gym, treadmill, and yoga can range from moderate to vigorous depending on how they are performed. Therefore, there is no clear-cut classification in this study between moderate to vigorous exercising since this study did not compare the differences in outcome measurements between these categories. The results of this study therefore fail to show the quantitative effectiveness of intensity and duration of activities on PC, PB, and PAQ.

Other potential factors may also affect PC, PB, and PAQ, e.g., perception of comfort and breathability may vary according to age, as younger individuals have more residual lung volume and lung capacities than elder adults [40]. For perceiving comfort and breathability, all individuals should have the same or a similar functional capacity of the parietal cortex which is an area of the brain responsible for sensory perception and integration, including the management of taste, hearing, sight, touch, and smell [41]. Its functional capacity changes due to age [41]. Perception is also strongly influenced by gender [42]. Body mass index (BMI) is also a factor, for the reason that it is linked to energy consumption and body metabolism. The shape of the participant’s face and the shape of the mask, hence the fitting, will likely affect the perception of breathability. In the future, well-designed cross-sectional prospective studies are required to further explore factors affecting the PC, PB, and PAQ of subjects during mask-wearing and the impact of PC, PB, and PAQ on their physiological and psychological aspects.

## Figures and Tables

**Table 1:** Participant characteristics.

	Gender		Age					Education Level		
	Male	Female	18–24	25–34	35–44	45–54	55–64	Secondary School	Bachelor	Post-Graduate
Count	61	43	11	37	39	13	4	17	71	16
%	58.7	41.3	10.6	35.6	37.5	12.5	3.8	16.3	68.3	15.4

**Table 2:** Types of face masks used by participants during daily activity and exercise.

Face Mask	Daily Activity Count (%)	Exercise Count (%)
Surgical	76 (73.1)	75 (72.1)
N95	9 (8.7)	6 (5.8)
KF94	16 (15.4)	14 (13.5)
Cotton/other	3 (2.9)	9 (8.7)

**Table 3:** Perceived comfort (PC) of wearing face masks.

Rating (Scale)	Daily Activity * Count (%)	Exercises * Count (%)
Very unsatisfied (−2)	12 (11.5)	23 (22.1)
Unsatisfied (−1)	17 (16.3)	44 (42.3)
Neutral (0)	39 (37.5)	22 (21.2)
Satisfied (1)	30 (28.8)	10 (9.6)
Very satisfied (2)	6 (5.8)	5 (4.8)

* Paired *t*-test, *p* < 0.01.

**Table 4:** Perceived breathability (PB) measure with the extent of disturbance to breath.

Rating (Scale)	Daily Activity * Count (%)	Exercise * Count (%)
Always (1)	15 (14.4)	27 (26.0)
Often (2)	14 (13.4)	41 (39.4)
Sometimes (3)	49 (47.1)	25 (24.0)
Seldom (4)	22 (21.2)	9 (8.7)
Never (5)	4 (3.8)	2 (1.9)

* Paired *t*-test, *p* < 0.01.

**Table 5:** Perceived air quality (PAQ) measured with perceived unpleasant breath odor and unpleasant sweat odor.

Rating (Scale)	Daily Activity		Exercise	
	Breath Odor * Count (%)	Sweat Odor ^ Count (%)	Breath Odor * Count (%)	Sweat Odor ^ Count (%)
Always (1)	11 (10.6)	8 (7.7)	18 (17.3)	22 (21.2)
Often (2)	12 (11.5)	13 (12.5)	35 (33.7)	29 (27.9)
Sometimes (3)	49 (47.1)	40 (38.5)	28 (26.9)	25 (24.0)
Seldom (4)	25 (24.0)	33 (31.7)	18 (17.3)	23 (22.1)
Never (5)	7 (6.7)	10 (9.6)	5 (4.8)	5 (4.8)

* Paired *t*-test, *p* < 0.01. ^ Paired *t*-test, *p* < 0.01.

## Data Availability

Some or all data that support the findings of this study are available from the corresponding author upon reasonable request. The data are not publicly available due to restrictions, e.g., containing information that could compromise the privacy of research participants.
